# Equivalent Survival between Gastric Large-Cell Neuroendocrine Carcinoma and Gastric Small-Cell Neuroendocrine Carcinoma

**DOI:** 10.3390/jcm12186039

**Published:** 2023-09-18

**Authors:** Zefeng Li, Hu Ren, Xiaojie Zhang, Chongyuan Sun, He Fei, Zheng Li, Chunguang Guo, Susheng Shi, Yingtai Chen, Dongbing Zhao

**Affiliations:** 1 Department of Pancreatic and Gastric Surgical Oncology, National Cancer Center/National Clinical Research Center for Cancer/Cancer Hospital, Chinese Academy of Medical Sciences and Peking Union Medical College, No. 17 PanjiayuanNanli, Chaoyang District, Beijing 100021, China; 2 Department of Pathology, National Cancer Center/National Clinical Research Center for Cancer/Cancer Hospital, Chinese Academy of Medical Sciences and Peking Union Medical College, No. 17 PanjiayuanNanli, Chaoyang District, Beijing 100021, China

**Keywords:** gastric neuroendocrine carcinoma, large-cell neuroendocrine carcinoma, small-cell neuroendocrine carcinoma, prognosis, survival outcome

## Abstract

Background: According to the 2019 World Health Organization (WHO) classification of gastric neuroendocrine neoplasms, gastric neuroendocrine carcinoma (GNEC) can be further divided into gastric large-cell neuroendocrine carcinoma (GLNEC) and gastric small-cell neuroendocrine carcinoma (GSNEC). Whether the prognoses of the two types have a discrepancy has long been disputed. Method: We collected patients diagnosed with GLNEC or GSNEC in the National Cancer Center of China between January 2000 and December 2020. The characteristics and survival outcomes were compared between the two groups. We further verified our conclusion using the SEER dataset. Results: A total of 114 GNEC patients, including 82 patients with GLNEC and 32 patients with GSNEC, have completed treatment in our hospital. Clinicopathologic differences were not observed between patients with GSNEC and GLNEC concerning the sex, age, body mass index, Charlson Comorbidity Index, tumor location, tumor size, stage, treatment received, the expression of neuroendocrine markers (CD56, Chromogranin A, synaptophysin), and score on the Ki-67 index. The 1-year, 3-year, and 5-year overall survival rates of GLNEC and GSNEC were 89.0%, 60.5%, and 52.4%, and 93.8%, 56.3%, and 52.7%, which showed no statistically significant differences. This result was confirmed further by using the SEER dataset after the inverse probability of treatment weighting. Conclusions: Although with different cell morphology, the comparison of prognosis between the GLNEC and GSNEC has no significant statistical difference.

## 1. Introduction

According to the 2019 World Health Organization (WHO) classification of gastric neuroendocrine neoplasms, gastric neuroendocrine carcinoma (GNEC), a kind of poorly differentiated and highly aggressive neuroendocrine neoplasm, can be further divided into two subclasses: gastric large-cell neuroendocrine carcinoma (GLNEC), characterized by a large atypical nuclei and abundant cytoplasm; and gastric small-cell neuroendocrine carcinoma (GSNEC), characterized by a high nuclear/cytoplasmic ratio and scant cytoplasm [1,2]. The origin of GNEC has not been fully determined [3,4]. Many scientists believe that some precursor cells in well-differentiated adenocarcinoma can differentiate into neuroendocrine cancer cells [5,6,7]. Whether GLNEC and GSNEC have different prognoses and should be treated separately remains unknown and needs to be elucidated. A better understanding of the prognosis of GNEC could influence treatment decisions, the follow-up period, and the patient’s psychological burden [8].

Results from previous population-based studies, however, have been inconsistent [2,9,10,11,12,13,14,15,16,17]. Some studies have reported a worse prognosis of GLNEC [9,11] or GSNEC [10,17], whereas others found no association between pathological morphology and survival outcomes. Most did not show the baseline characteristics or they contained neuroendocrine carcinoma of other organs. One analysis conducted by Nam Young Choi, which included only GNEC patients and showed baseline characteristics, suggested that the prognosis of GLNEC was worse than GSNEC, but the sample was small with only 23 GLNEC and 13 GSNEC patients [9].

As such, we conducted the most comprehensive study in the National Cancer Center of China to compare the prognoses of GLNEC and GSNEC, and verified our conclusion using the SEER Database, in the hope of providing more information for clinical diagnosis and treatment for GNEC.

## 2. Method

### 2.1. Patients and Study Design

We conducted a clinical retrospective cohort study of GNEC. The patients diagnosed with GNEC at the National Cancer Center of China between January 2000 and December 2020 and treated in the hospital were included in this study. The diagnosis of GNEC was confirmed by morphology and positive immunohistochemical staining with three neuroendocrine markers, synaptophysin SYN (cat. no. ZA-0506), chromogranin A CgA (cat. no. ZM-0076), and CD56 (cat. no. ZM-0057) [18]. According to the degree of positive cells, the staining results were evaluated as follows: <5%, negative; 5% to 50%, focal; and >50%, diffuse [2]. The diagnosis of GNEC was confirmed by two pathologists individually, using the 2019 World Health Organization (WHO) classification of gastric neuroendocrine neoplasms for histopathologic evaluation [1]. The exclusion criteria were as follows: (1) patients died within one month of the operation (2 cases for postoperative hemorrhage); (2) patients had no details about cancer cell type (31 cases); and (3) patients had mixed large-cell and small-cell GNEC (1 case) [19]. Patients with GNEC were then subdivided into GLNEC and GSNEC groups according to the different cytomorphologic features depicted in the WHO classification [1,20]. We retrieved the patients’ sex, age, body mass index (BMI), comorbidity status (the Charlson Comorbidity Index (CCI) gives each medical condition, such as heart disease, diabetes, kidney disease, and cancer, a score ranging from 1 to 6 according to its impact on patient mortality [21]), tumor location, stage, treatment received (surgery, chemotherapy, or radiotherapy), neuroendocrine markers (synaptophysin (SYN), chromogranin A (CgA), and CD56), and score on the Ki-67 index. The TNM stage was re-evaluated according to the American Joint Committee on Cancer (AJCC) 8th edition staging definition for gastric cancer [22]. According to a previous study about GNEC, we selected 60 years, 6 cm, and 70% as the cutoff values for age, tumor size, and Ki-67 index, respectively [14]. We divided patients with GNEC into 3 groups (underweight < 18.5, normal/overweight 18.5–27.9, and obese ≥ 28) by BMI according to the guidelines for prevention and control of overweight and obesity in Chinese adults [23]. Surgery received by all patients in our center was radical, D2, R0 gastrectomy, except one stage IV patient who received gastrojejunostomy for pyloric obstruction and was assigned to the non-surgery group. All patients were followed up every 3 or 6 months, via re-examinations in the outpatient clinic or by telephone, until mortality due to any reason or loss of follow-up. Overall survival (OS) time was measured from the date of diagnosis to the date of death or the date of the last follow-up (updated on 1 March 2022).

We used the Surveillance, Epidemiology, and End Results (SEER) Database (www.seer.cancer.gov (accessed on 1 November 2021)) to conduct a retrospective review of patients with GNEC in the United States. We used the database ‘SEER Research Plus Data, 18 Registries, November 2020 Sub (2000–2018)’ to screen the cases of GNEC. Patients were identified according to ICD-0-3 histology codes for neuroendocrine carcinoma as “8013 (Large-cell neuroendocrine carcinoma) and 8041 (Small-cell carcinoma, NOS)”, and site-specific codes for gastric tumors as “C16.0–C16.9, stomach”. The exclusion criteria were: cases with unknown tumor size, cases with unknown TNM stage, cases with unknown treatment details, and cases without follow-up information. We collected the patients’ demographic (age and sex), diagnostic (tumor site, tumor size, and tumor stage), treatment (surgery, radiation, or chemotherapy), and follow-up information. We re-evaluated the TNM stage in line with the 8th AJCC staging definition for gastric cancer [22]. OS was defined from the date of diagnosis to the date of death or last follow-up visit. The data obtained from the SEER database in this study followed the SEER data use agreement (ID: 17851-Nov2020).

### 2.2. Statistical Analysis

Categorical variables were presented as numbers and percentages and compared between subgroups using χ2 tests or the Fisher exact test appropriately. We used the inverse probability of treatment weighting (IPTW) method to adjust the observed differences in baseline covariates between the large-cell and small-cell groups in the SEER dataset to account for the selection bias. Age, sex, tumor location, tumor size, tumor stage, and treatment received were included in constructing the logistic model. Survival outcomes were illustrated with the Kaplan–Meier method and compared using log-rank tests. The variables with a hazard ratio (HR) < 0.1 in the univariate analyses were included in the multivariable Cox regression model to identify factors independently associated with the prognosis. HR and 95% confidence interval (CI) were used to estimate survival predictors. All statistical analyses were performed using R version 4.0.4 (R Project for Statistical Computing) and SPSS (version 23; IBM Corp, Armonk, NY, USA), with *p* values < 0.05 considered statistically significant.

## 3. Results

### 3.1. The National Cancer Center of China Cohort

**Figure 1:**
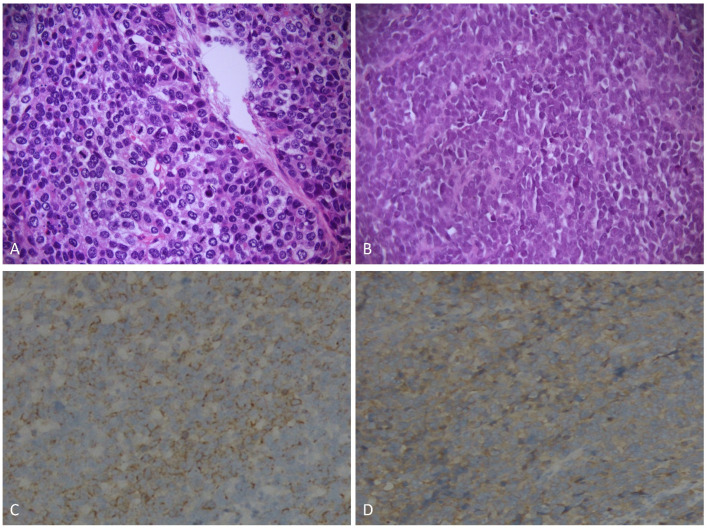
Histological Findings of the Gastric Neuroendocrine Carcinoma. (**A**): Gastric Large-cell Neuroendocrine Carcinoma (hematoxylin-eosin (HE), ×400). (**B**): Gastric Small-cell Neuroendocrine Carcinoma (HE, ×400). (**C**): Positive Immunohistochemical Staining for Chromogranin A (×400). (**D**): Positive Immunohistochemical Staining for Synaptophysin (×400).

A total of 114 patients with GNEC were enrolled. The characteristics of the patients with GNEC in our hospital are shown in Table 1. Most GNEC patients were male (83.3%), with a mean age of 61.6 years. And 69.3% of GNEC patients were assessed as 0 score according to CCI. Moreover, 71.1% of GNEC were located in the upper stomach, with a mean tumor size of 5.6 cm. The I-II, III, and IV stage of the patients with GNEC were 29 (25.5%), 70 (61.4%), and 15 (13.2%), respectively. A large proportion of the patients received surgery (89.5%) and chemotherapy (75.4%), but only four patients received radiotherapy. The patients with positive CD56, CgA, and SYN were 72.6%, 81.2%, and 96.2%, respectively. And 56.1% of patients had tumors with Ki-67 > 70%. There were 82 cases of GLNEC and 32 cases of GSNEC. As Figure 1 shows, GLNEC was large with a distinct cell border, low ratio of nuclear to cytoplasm, and evident nucleolus, while GSNEC was small with an indistinct cell border, high ratio of nuclear to cytoplasm, and inconspicuous or even absent nucleolus. The above variables in the two groups had no statistically significant difference.

**Figure 2:**
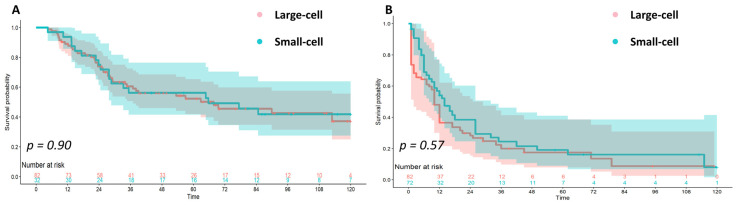
Kaplan–Meier Survival Curves for Patients with Gastric Neuroendocrine Carcinoma ((**A**): in the National Cancer Center of China Cohort; (**B**): in the Weighted SEER Population).

The follow-up time range was 4–230 months. The 1-year, 3-year, and 5-year OS were 90.4%, 59.2%, and 52.5%. We then compared the prognoses of GLNEC and GSNEC (Figure 2A). The 1-year, 3-year, and 5-year OS among the GLNEC group and GSNEC group were 89.0%, 60.5%, and 52.4%, and 93.8%, 56.3%, and 52.7% (Table 1). There was no significant statistical difference between the two subgroups concerning OS (*p* = 0.90). We then conducted the Cox analysis, and the results showed that tumor cell type is not an independent prognostic factor (Table 2). Receiving chemotherapy and having earlier stage tumors were identified as independent prognostic factors associated with better prognosis in GNEC. Also, having advanced GNEC was an independent prognostic factor related to poorer prognosis for both GLNEC and GSNEC. Having comorbidity indicated a poorer prognosis for GLNEC and immunohistochemistry with diffuse positive CgA indicated a poorer prognosis for GSNEC. We then compared the prognoses of GLNEC and GSNEC in the subgroups, all of which showed similar results (all *p* > 0.05, Supplementary Table S1).

### 3.2. The SEER Cohort

To further verify the conclusion in our hospital cohort, we compared the prognoses of GLNEC and GSNEC in the SEER dataset. A total of 79 GNEC patients were identified, including 38 patients with GLNEC and 41 patients with GSNEC. Supplementary Table S2 shows the baseline characteristics between the two groups. Compared with patients with GLNEC, patients with GSNEC were younger but had more distant metastasis, and a lower proportion of them had received surgery. As a result, the IPTW method was conducted to balance the bias between the GLNEC and GSNEC. After IPTW, all variables between the two groups were comparable, with all *p* > 0.1 (Supplementary Table S2). The weighted Kaplan–Meier curve also shows that there was no significant statistical difference concerning OS between the two groups (Figure 2B, *p* = 0.57).

## 4. Discussion

We conducted the research with a relatively large sample and the most comprehensive variables to compare the differences between GLNEC and GSNEC, and found there are no significant differences in clinical characteristics and prognoses between GLNEC and GSNEC. Classifying GSNEC and GLNEC in the same category is reasonable.

GNEC is a rare malignant disease that is increasing in incidence, and has therefore attracted growing attention [24]. However, because of its rarity, few studies have compared GLNEC and GSNEC. Studies are also limited by the small number of suitable patients. In lung cancer, which has a higher incidence, the prognosis of large-cell neuroendocrine carcinoma of the lung is regarded as worse than small-cell lung carcinoma, and the treatment for the two types of lung neuroendocrine carcinoma is not completely identical [25]. In the 2019 WHO classification, GNEC was divided into GLNEC and GSNEC. However, no further distinction was made in the clinical guidelines for their treatment, and little is known about the differences in their prognoses [26]. Therefore, we designed and conducted this research.

In our center, we found the incidence of GLNEC outnumbered GSNEC by two to one, which was similar to the findings of Matsui K [27] and Nam Young Choi [9]. In other previous research, the proportion of GLNEC was higher than GSNEC but with a different ratio (1.5–5:1) [2,10,11,12,13,15,23]. However, in the SEER dataset, the incidence ratio of GLNEC and GSNEC seemed to be 1:1. Considering most of the above studies were based on the East Asian population, the reason for this phenomenon needs further study to determine if there a racial disparity or if it is the result of a limited sample size. We did not find a significant difference in clinicopathologic characteristics between GLNEC and GSNEC. They were both most often diagnosed in men around 60 years old and more likely to be located in the upper portion of the stomach, which corresponds with Michihiro Ishida’s findings [2]. Both GLNEC and GSNEC were usually locally advanced when diagnosed, which shows the highly malignant biological behavior of GLNEC and GSNEC, and the treatment modalities are essentially the same in our center. Surgical treatment is the major means of combined modality therapy for operable GLNEC and GSNEC. There was also no difference found in neuroendocrine markers expression (SYN, CgA, and CD56). We confirmed that SYN was the most sensitive marker (the positive rate was 96.2%) in both GLNEC and GSNEC, as Michihiro Ishida reported (Table 1) [2].

Jianxian Lin suggested that the 5-year OS of resectable GNEC was 50.5% in a multicenter cohort study [28]. Although we included 13.2% stage IV patients, most of whom (11/15) did not receive surgery, the 5-year OS was 52.5% in our center. The reason might be that patients in our center were much younger (range: 34–84, mean: 61) with less comorbidity. We then found that early stage and receiving chemotherapy were independent risk factors associated with a better prognosis, whereas GNEC cell morphology, neuroendocrine markers, and Ki-67 index were not (Table 2). As previous studies reported, chemotherapy played a crucial role in the treatment of GNEC, not only in stage IV GNEC with a palliative aim but also in the resectable GNEC in the form of perioperative chemotherapy [29,30]. However, we did not find this phenomenon in either GLNEC or GSNEC. The reason might be the heterogeneity of the chemotherapy regime or the limited sample size. In addition, having no comorbidity was related to a better prognosis in GLNEC besides the early stage. However, in GLNEC, having distant metastasis was not regarded as an independent prognostic factor related to worse survival with a *p* > 0.05 (HR 3.245 (0.981, 10.738). We suspect that some GNEC patients sensitive to chemotherapy could achieve long-term survival even if distant metastasis occurs.

In GSNEC, patients with focal CgA expression had a better prognosis (HR 0.052, 95%CI 0.007–0.390). In the subsequent subgroup analysis (Supplementary Table S1), patients with GSNEC with focal CgA expression seemed to have a better prognosis than patients with GLNEC (5-year OS: 84.6% vs. 52.6%, *p* = 0.056). Yujie Deng et al. found no significant correlation between survival and expression of SYN, CgA, or Ki-67 in GNEC [13]. But they did not distinguish between GLNEC or GSNEC. Whether CgA expression could be a prognostic factor in predicting GSNEC prognosis needs more study. We found no survival difference between GLNEC and GSNEC in all subgroups. We used the SEER dataset to further validate the conclusion. To balance the observed differences in baseline covariates between GLNEC and GSNEC, the IPTW method was used and the results confirmed that the two types of GNEC had no statistically significant difference in the prognosis (Figure 2B).

In lung cancer, next-generation sequencing studies have shown that large-cell neuroendocrine carcinoma can be further subdivided into two mutually exclusive groups based on their mutational patterns: the small-cell carcinoma-like type and the non-small-cell carcinoma-like type [31,32]. It was speculated in the following study, but not confirmed, that molecular subtypes have a different response to chemotherapy regimens, with the small-cell carcinoma-like subtype more favorable toward etoposide and the non-small-cell carcinoma-like subtype toward gemcitabine/taxanes [33,34]. Regarding the chemotherapy regimen for advanced large-cell neuroendocrine carcinoma of the lung, treatment similar to small-cell carcinoma is more appropriate than non-small-cell lung carcinoma [35]. In the pancreas, small-cell and large-cell neuroendocrine carcinoma are genetically similar to each other. Management of large-cell neuroendocrine carcinoma of the pancreas may benefit from therapeutic approaches broadly similar to that of small-cell carcinomas [36]. However, there is a lack of relevant genetic studies in GNEC, which needs further study to improve the survival outcomes of patients with GNEC in the future. Studies of molecular genetics are still warranted to clarify if there is a need to take different treatments when facing GLNEC or GSNEC. Until then, it would be wise to treat the GLNEC and GSNEC as a single entity in clinical practice.

There are some limitations in our study, including its retrospective and single-center nature. Moreover, after dividing into GLNEC and GSNEC, it is quite difficult to interpret whether some phenomena are coincidental, such as GSNEC patients with focal CgA expression having a relatively good prognosis, because of the small sample size. They still need to be confirmed by large-sample, multicenter trials.

## 5. Conclusions

As far as we know, this study is the most comprehensive and the largest one comparing the prognoses between GLNEC and GSNEC. There were no apparent differences in clinical characteristics and survival outcomes between GLNEC and GSNEC. Therefore, we speculated that GLNEC and GSNEC might not have biologically different characteristics, except for cell size and morphologic features. Studies of molecular genetics are still warranted to explore the mechanisms of carcinogenesis in the GLNEC and GSNEC. Until other hard evidence emerges, they may be regarded as a single entity in clinical practice.

## Figures and Tables

**Table 1:** Baseline Clinicopathologic Characteristics of Patients with Gastric Large-Cell Neuroendocrine Carcinoma and Gastric Small-Cell Neuroendocrine Carcinoma in the National Cancer Center of China Cohort.

Characteristic	All *N* = 114 (%)	Large-Cell *N* = 82 (%)	Small-Cell *N* = 32 (%)	*p* Value
Sex				0.64
Male	95 (83.3)	67 (81.7)	28 (87.5)	
Female	19 (16.7)	15 (18.3)	4 (12.5)	
Age, years				1.00
<60	50 (43.9)	36 (43.9)	14 (43.8)	
≥60	64 (56.1)	46 (56.1)	18 (56.2)	
BMI				0.37
<18.5	8 (7.0)	6 (7.3)	2 (6.2)	
18.5–27.9	91 (79.8)	63 (76.8)	28 (87.5)	
≥28	15 (13.2)	13 (15.9)	2 (6.2)	
CCI				0.13
0	79 (69.3)	53 (64.6)	26 (81.2)	
1–5	35 (30.7)	29 (35.4)	6 (18.8)	
Tumor location				0.16
Upper	81 (71.1)	56 (68.3)	25 (78.1)	
Middle	14 (12.3)	9 (11.0)	5 (15.6)	
Lower	19 (16.7)	17 (20.7)	2 (6.2)	
Tumor size, cm				0.52
<6	64 (56.1)	44 (53.7)	20 (62.5)	
≥6	50 (43.9)	38 (46.3)	12 (37.5)	
Stage				0.80
I-II	29 (25.5)	22 (26.9)	7 (21.9)	
III	70 (61.4)	50 (61.0)	20 (62.5)	
IV	15 (13.2)	10 (12.2)	5 (15.6)	
Surgery				0.27
No	12 (10.5)	7 (8.5)	5 (15.6)	
Yes	102 (89.5)	75 (91.5)	27 (84.4)	
Chemotherapy				0.10
No	28 (24.6)	24 (29.3)	4 (12.5)	
Yes	86 (75.4)	58 (70.7)	28 (87.5)	
Chemotherapy regimen				0.91
Platinum based	46 (53.5)	32 (55.2)	14 (50)	
Non-platinum based	22 (25.6)	15 (25.9)	7 (25)	
Radiotherapy				1.00
No	110 (96.5)	79 (96.3)	31 (96.9)	
Yes	4 (3.5)	3 (3.7)	1 (3.1)	
CD56				0.06
Diffuse	48 (50.5)	32 (45.1)	16 (66.7)	
Focal	21 (22.1)	15 (21.1)	6 (25.0)	
Negative	26 (27.3)	24 (33.8)	2 (8.3)	
Chromogranin A				0.99
Diffuse	45 (42.5)	35 (45.5)	10 (34.5)	
Focal	41 (38.7)	28 (36.4)	13 (44.8)	
Negative	20 (18.9)	14 (18.2)	6 (20.7)	
Synaptophysin				0.29
Diffuse	76 (71.0)	59 (74.7)	17 (60.7)	
Focal	27 (25.2)	18 (22.8)	9 (32.1)	
Negative	4 (3.7)	2 (2.5)	2 (7.1)	
Ki-67				0.11
<70%	42 (36.8)	27 (36.5)	15 (46.9)	
≥70%	64 (56.1)	47 (63.5)	17 (53.1)	
OS				0.90
1-year OS (%)	90.4 (84.9, 95.9)	89.0 (82.1, 95.9)	93.8 (85.4, 100.0)	
3-year OS (%)	59.2 (50.0, 68.4)	60.5 (49.5, 71.5)	56.3 (39.1, 73.5)	
5-year OS (%)	52.5 (42.9, 62.1)	52.4 (40.8, 64.0)	52.7 (35.3, 70.1)	
Median OS (months)	65.7 (38.5, 93.0)	67.8 (22.4, 113.2)	65.7 (5.2, 126.3)	

BMI: body mass index; CCI: Charlson Comorbidity Index; OS: overall survival.

**Table 2:** Univariable and Multivariable Cox Regression Analyses of Factors Associated with Overall Survival of Patients with Gastric Neuroendocrine Carcinoma in the National Cancer Center of China Cohort.

Characteristic	All		Large-Cell		Small-Cell	
	Univariable	Multivariable	Univariable	Multivariable	Univariable	Multivariable
Sex						
Male	Reference		Reference		Reference	
Female	1.041 (0.527, 2.056)		0.800 (0.353, 1.811)		2.130 (0.610, 7.348)	
Age, year						
<60	Reference		Reference		Reference	
≥60	1.069 (0.643, 1.777)		1.273 (0.492, 3.290)		0.659 (0.151, 2.868)	
BMI						
<18.5	Reference		Reference		Reference	
18.5–27.9	0.669 (0.266, 1.685)		0.640 (0.225, 1.822)		0.900 (0.119, 6.926)	
≥28	0.770 (0.252, 2.358)		0.867 (0.253, 2.969)		0.570 (0.036, 9.144)	
CCI						
0	Reference	Reference	Reference	Reference	Reference	
1–5	1.618 (0.950, 2.757) *	1.584 (0.924, 2.717) *	1.915 (1.028, 3.567) **	1.968 (1.050, 3.688) **	1.130 (0.326, 3.914)	
Tumor location						
Proximal	Reference		Reference		Reference	Reference
Middle	0.835 (0.376, 1.855)		0.461 (0.139, 1.530)		1.828 (0.593, 5.640)	0.594 (0.105, 3.361)
Distal	0.647 (0.305, 1.373)		0.624 (0.273, 1.426)		0.707 (0.092, 5.445) *	0.157 (0.015, 1.626)
Tumor size, cm						
<6	Reference		Reference		Reference	
≥6	1.057(0.625, 1.787)		1.276 (0.686, 2.374)		0.672 (0.236, 1.913)	
Stage						
I-II	Reference		Reference		Reference	
III	2.716 (1.322, 5.579) ***	3.631 (1.722, 7.658) ***	3.198 (1.325, 7.719) **	3.256 (1.348, 7.868) ***	1.827 (0.515, 6.487)	6.908 (1.278, 37.333) **
IV	2.729 (1.050, 7.094) **	4.318(1.555, 11.991) ***	3.169 (0.962, 10.446) *	3.245 (0.981, 10.738) *	1.934 (0.387, 9.661)	23.688 (1.369, 409.966) **
Surgery						
No	Reference		Reference		Reference	
Yes	0.846 (0.364, 1.969)		0.866 (0.266, 2.823)		0.819 (0.238, 2.821)	
Chemotherapy						
No	Reference	Reference	Reference		Reference	
Yes	0.597 (0.346, 1.029) *	0.416 (0.231, 0.747) ***	0.589 (0.311, 1.113)		0.602 (0.198, 1.829)	
Radiotherapy						
No	Reference		Reference		Reference	
Yes	0.646 (0.157, 2.651)		0.477 (0.065, 3.477)		0.987 (0.129, 7.532)	
CD56						
Diffuse	Reference		Reference		Reference	
Focal	1.196 (0.560, 2.554)		1.675 (0.725, 3.869)		0.171 (0.023, 1.277) *	0.362 (0.023, 5.583)
Negative	1.207 (0.618, 2.357)		1.087 (0.490, 2.409)		0.548 (0.119, 2.517)	0.793 (0.080, 7.819)
Chromogranin A						
Diffuse	Reference		Reference		Reference	Reference
Focal	0.616 (0.316, 1.202)		0.902 (0.401, 2.029)		0.199 (0.053, 0.748) **	0.052(0.007, 0.390) ***
Negative	0.694 (0.350, 1.374)		0.650 (0.280, 1.508)		0.750 (0.234, 2.404)	0.469(0.093, 2.350)
Synaptophysin						
Diffuse	Reference		Reference		Reference	
Focal	1.607 (0.373, 6.911)		1.190 (0.154, 9.180)		1.265 (0.145, 11.033)	
Negative	1.184 (0.285, 4.922)		0.592 (0.079, 4.425)		2.363 (0.300, 18.595)	
Ki-67						
<70%	Reference		Reference		Reference	
≥70%	1.234 (0.735, 2.073)		1.130 (0.591, 2.158)		1.603 (0.642, 3.999)	
Type						
Large-cell	Reference					
Small-cell	0.989 (0.597, 1.719)					

BMI: body mass index; CCI: Charlson Comorbidity Index; * <0.1, ** <0.05, *** <0.01.

## Data Availability

The datasets obtained during the present study are not publicly available because of privacy/ethical restrictions, but are available from the corresponding author upon reasonable request.

## Supplementary Materials

The following supporting information can be downloaded at: https://www.mdpi.com/article/10.3390/jcm12186039/s1, Table S1: Survival outcomes of Patients with Gastric Large-Cell Neuroendocrine Carcinoma and Gastric Small-Cell Neuroendocrine Carcinoma in the subgroups in the National Cancer Center of China Cohort; Table S2: Baseline Clinicopathologic Characteristics of Patients with Gastric Large-Cell Neuroendocrine Carcinoma and Gastric Small-Cell Neuroendocrine Carcinoma in the SEER Database.
