# Supplementary material for: Equivalent Survival between Gastric Large-Cell Neuroendocrine Carcinoma and Gastric Small-Cell Neuroendocrine Carcinoma
Source: J Clin Med. 2023 Sep 18;12(18):6039. doi: 10.3390/jcm12186039 (PMC10531653; doi:10.3390/jcm12186039)
Supplement: Supplementary file 1 [file jcm-12-06039-s001.zip › jcm-2569792-supplementary.pdf]

Supplementary Table S1. Survival outcomes of Patients with Gastric Large-Cell Neuroendocrine Carcinoma and Gastric Small-Cell Neuroendocrine Carcinoma in the subgroups in the National Cancer Center of China Cohort

Supplementary Table S2. Baseline Clinicopathologic Characteristics of Patients with Gastric Large-Cell Neuroendocrine Carcinoma and Gastric Small-Cell Neuroendocrine Carcinoma in the SEER Database

Supplementary Table S1. Survival outcomes of Patients with Gastric Large-Cell Neuroendocrine Carcinoma and Gastric Small-Cell Neuroendocrine Carcinoma in the National Cancer Center of China Cohort

	**Large-Cell**			**Small-Cell**			
**Characteristic**	**1 year-OS (%)**	**3 year-OS (%)**	**5 year-OS (%)**	**1 year-OS (%)**	**3 year-OS (%)**	**5 year-OS (%)**	**P**
**Sex**							
**Male**	82.1	56.4	40.9	92.9	57.1	57.1	0.780
**Female**	100	71.4	71.4	75.0	50.0	NA	0.177
**Age, year**							
**<60**	94.4	54.4	50.8	92.9	57.1	49.0	0.791
**≥60**	82.6	62.5	53.0	94.4	55.6	55.6	0.772
**BMI**							
**<18.5**	50.0	50.0	50.0	83.3	50.0	50.0	0.919
**18.5-27.9**	90.5	63.6	53.2	92.9	53.6	53.6	0.734
**≥28**	84.6	49.9	49.9	100	100	100	0.439
**Tumor location**							
**Upper**	87.5	57.4	45.5	92.0	56.0	51.3	0.433
**Middle**	88.9	64.8	64.9	100	40.0	40.0	0.159
**Lower**	88.2	70.1	70.1	100	100	100	0.896
**Tumor size, cm**						0.900	
**<6**	93.2	59.0	50.5	90.0	55.0	55.0	0.742
**≥6**	82.4	61.0	52.4	100	70.0	70.0	0.330
**Stage**							
**I-II**	95.5	86.4	69.8	100	71.4	71.4	0.679
**III**	90.0	50.7	45.7	90.0	55.0	55.0	0.571
**IV**	70.0	45.0	NA	100	40.0	NA	0.954

**Surgery**							
**No**	71.4	57.1	NA	100	40.0	NA	0.834
**Yes**	90.7	60.8	52.0	92.6	59.3	59.3	0.908
**Chemotherapy**							
**No**	75.0	52.3	41.2	100	50.0	50.0	0.966
**Yes**	93.1	64.1	57.9	92.9	57.1	57.1	0.733
**Radiotherapy**							
**No**	88.6	60.4	51.9	93.5	54.8	54.8	0.982
**Yes**	100	66.7	NA	NA	NA	NA	0.564
**CD56**							
**Diffuse**	93.8	62.3	49.1	93.8	43.8	43.8	0.245
**Focal**	80.0	53.3	46.7	83.3	66.7	66.7	0.139
**Negative**	87.5	61.8	61.8	100	50.0	NA	0.128
**Chromogranin A**							
**Diffuse**	97.1	62.6	52.8	80.0	40.0	40.0	0.243
**Focal**	78.6	56.7	52.6	100	84.6	84.6	0.056
**Negative**	93.8	62.3	49.1	93.8	43.8	43.8	0.245
**Synaptophysin**							
**Diffuse**	91.5	62.2	58.1	88.2	47.1	47.1	0.233
**Focal**	77.8	50.0	29.2	100	66.7	66.7	0.059
**Negative**	100	50.0	NA	100	100	100	0.317
**Ki67**							
**<70%**	92.6	65.8	52.8	93.8	68.8	68.8	0.552
**≥70%**	85.5	57.7	52.2	93.8	43.8	43.8	0.626

BMI: body mass index; CCI: Charlson Comorbidity Index; OS: overall survival

Supplementary Table S2. Baseline Clinicopathologic Characteristics of Patients with Gastric Large-Cell Neuroendocrine Carcinoma and Gastric Small-Cell Neuroendocrine Carcinoma in the SEER Database

Characteristic	Unweighted Study Population, No. (%)			Weighted Study Population, No.		
	Large-Cell N=38	Small-Cell N=41	P value	Large-Cell N=82.4	Small-Cell N=72.3	P value
**Sex**			0.780			0.734
**Male**	28 (73.7)	28 (68.3)		63.9	53.4	
**Female**	10 (26.3)	13 (31.7)		18.5	19.0	
**Age, year**			0.024			0.971
**<60**	6 (15.8)	17 (41.5)		27.3	23.6	
**≥60**	32 (84.2)	24 (58.5)		55.1	48.8	
**Tumor location**			0.546			0.732
**Proximal**	14 (36.8)	23 (56.1)		34.1	36.0	
**Middle**	13 (34.2)	10 (24.4)		32.6	18.7	
**Distal**	6 (15.8)	4 (9.8)		8.4	7.5	
**Mix**	3 (7.9)	2 (4.9)		3.2	2.2	
**Unknown**	2 (5.3)	2 (4.9)		4.1	8.0	
**Tumor size, cm**			0.582			0.900
**<6**	18 (47.4)	23 (56.1)		49.3	42.0	
**≥6**	20 (52.6)	18 (43.9)		33.1	30.3	
**Stage**			0.035			0.915
**I**	6 (15.8)	10 (24.4)		14.5	15.1	
**II**	8 (21.1)	6 (14.6)		14.4	16.1	
**III**	15 (39.5)	6 (14.6)		19.6	12.7	
**IV**	9 (23.7)	19 (46.3)		33.9	28.5	

**Surgery**			<0.001			0.852
**No**	9 (23.7)	27 (65.9)		38.0	35.4	
**Yes**	29 (76.3)	14 (34.1)		44.4	36.9	
**Chemotherapy**			0.143			0.405
**No**	14 (36.8)	8 (19.5)		29.7	17.3	
**Yes**	24 (63.2)	33 (80.5)		52.7	55.0	
**Radiotherapy**			0.780			0.532
**No**	28 (73.7)	28 (68.3)		59.8	46.5	
**Yes**	10 (26.3)	13 (31.7)		22.6	25.9	
